# Using Voice Biomarkers to Classify Suicide Risk in Adult Telehealth Callers: Retrospective Observational Study

**DOI:** 10.2196/39807

**Published:** 2022-08-15

**Authors:** Ravi Iyer, Maja Nedeljkovic, Denny Meyer

**Affiliations:** 1 Centre for Mental Health Swinburne University of Technology Hawthorn Australia

**Keywords:** voice biometrics, suicide prevention, machine learning, telehealth, suicide, telehealth, risk prediction, prediction model, voice biomarker, mental health

## Abstract

**Background:**

Artificial intelligence has the potential to innovate current practices used to detect the imminent risk of suicide and to address shortcomings in traditional assessment methods.

**Objective:**

In this paper, we sought to automatically classify short segments (40 milliseconds) of speech according to low versus imminent risk of suicide in a large number (n=281) of telephone calls made to 2 telehealth counselling services in Australia.

**Methods:**

A total of 281 help line telephone call recordings sourced from On The Line, Australia (n=266, 94.7%) and 000 Emergency services, Canberra (n=15, 5.3%) were included in this study. Imminent risk of suicide was coded for when callers affirmed intent, plan, and the availability of means; level of risk was assessed by the responding counsellor and reassessed by a team of clinical researchers using the Columbia Suicide Severity Rating Scale (=5/6). Low risk of suicide was coded for in an absence of intent, plan, and means and via Columbia suicide Severity Scale Ratings (=1/2). Preprocessing involved normalization and pre-emphasis of voice signals, while voice biometrics were extracted using the statistical language r. Candidate predictors were identified using Lasso regression. Each voice biomarker was assessed as a predictor of suicide risk using a generalized additive mixed effects model with splines to account for nonlinearity. Finally, a component-wise gradient boosting model was used to classify each call recording based on precoded suicide risk ratings.

**Results:**

A total of 77 imminent-risk calls were compared with 204 low-risk calls. Moreover, 36 voice biomarkers were extracted from each speech frame. Caller sex was a significant moderating factor (*β*=–.84, 95% CI –0.85, –0.84; *t*=6.59, *P*<.001). Candidate biomarkers were reduced to 11 primary markers, with distinct models developed for men and women. Using leave-one-out cross-validation, ensuring that the speech frames of no single caller featured in both training and test data sets simultaneously, an area under the precision or recall curve of 0.985 was achieved (95% CI 0.97, 1.0). The gamboost classification model correctly classified 469,332/470,032 (99.85%) speech frames.

**Conclusions:**

This study demonstrates an objective, efficient, and economical assessment of imminent suicide risk in an ecologically valid setting with potential applications to real-time assessment and response.

**Trial Registration:**

Australian New Zealand Clinical Trials Registry ACTRN12622000486729; https://www.anzctr.org.au/ACTRN12622000486729.aspx

## Introduction

Suicide remains the 4th leading cause of death among 15- to 45-year-olds internationally [1]. However, traditional risk factor–based assessment has failed to identify suicide risk in a targeted and timely manner [2]. There has been historically a poor understanding of which risk factors contribute most to identifying an escalation in suicide risk [2]. This has led to calls for alternative approaches to evaluation, coupled with more powerful means of analysis [3].

Suicide risk assessment using voice biomarkers holds significant promise. Several candidate voice biomarkers have been identified that discriminate accurately between low and high risk of suicide, including timing and prosody–based features [4]. When combined with high-powered forms of statistical analysis (eg, machine learning), voice biomarkers offer an objective, unobtrusive, and economically feasible approach for this purpose.

In a promising study by Pestian and colleagues [5], an accuracy of 85% was obtained using a range of voice biomarkers that classified 379 calls according to the 3 categories of high risk of suicide, mentally ill without prior history of suicidal ideation, and healthy controls. However, the classification was obtained using support vector machines, a powerful machine learning approach that can analyze nonlinear data, but for which post hoc interpretability is unavailable [6]. Thus, with a support vector machine, it is difficult to understand which voice biomarkers are important and which are not. These important considerations will be addressed in our new study.

Souririajan and colleagues [7] replicated the Pestian and colleagues [5] study with 94 US veterans, meeting the criteria for Gulf War syndrome measured at months 0, 1, 2, 3, 6, and 12 after recruitment. A range of voice biomarkers provided only modest discrimination between low and high risk of suicide (area under the receiver operating characteristic curve=0.64) [7]. However, male Gulf War veterans, who formed the majority of the sample (80.0%), are at lower risk of suicide than the general population [8], and reliance upon item 9 of the Patient Health Questionnaire alone (“Thoughts that you’d be better off dead, or thoughts of hurting yourself in some way?”) is associated with higher rates for false positives when compared with the more comprehensive Columbia Suicide Severity Rating Scale used by Pestian and colleagues [5].

Attempts have been made by a number of international jurisdictions to codify a hierarchy of patient suicide risk and appropriate response. Following the UK guidelines, Victoria, Australia has developed the Statewide Mental Health Triage Scale [9]. Seven levels of risk are defined, with “current actions endangering self” afforded the highest level of risk, followed by very high risk of imminent harm, high risk, moderate risk, low risk, referral required, and advice or information provision at the lowest level of risk. The second highest category, very high risk, specifies acute suicidal ideation accompanied by clear plan and means. Neither Pestian and colleagues [5] nor Sourirajan [7] clearly indicated which level of risk was being targeted in their studies. In this new study, we are targeting low risk and below compared with very high risk of imminent harm.

Furthermore, the studies by Pestian and colleagues [5] and Sourirajan [7] lack translation into real-world settings. Based on their studies, high levels of accuracy seem plausible only when participants are recruited from inpatient services [10,11], interviewed under lab conditions, and risk of suicide is assumed to remain static over time. To extend the generalizability of these findings, participants need to be recruited from ecologically valid settings and assessed when elevation in suicide risk occurs.

The help line services we partnered with in this study represent ecologically valid settings. Help line services have played an important role in early detection and response to suicide risk in the community since the early 1950s [12]. In recent years, help line services have witnessed a significant increase in the volume of suicide-related presentations resulting from the COVID-19 pandemic [12,13]. Help line services support important avenues of suicide detection and prevention by providing equitability of access, promotion of disclosures and trust, and supplementation of traditional forms of health care [14]. However, suicide assessment via telehealth is challenged by the absence of nonverbal cues, by time limitations, and by the reticence of some callers to verbally express suicidal intent [15].

Where there is reasonable suspicion that the caller may have taken actions to endanger themselves, emergency management protocols can be triggered. This typically involves dispatch of police and ambulance to perform a welfare check. However, scarce emergency resources can also be dispatched when the caller is not at imminent risk, thus potentially diverting life-saving services from other emergencies. Alternatively, there is the threat of a serious risk of harm when imminent risk of suicide is not detected and therefore not responded to. These unfortunate high-stake scenarios can result in high-pressure work environments that can adversely affect service providers and the individual affected, making it critically important that assessments of imminent suicide risk are as close to 100% correct as possible.

Artificial intelligence has the potential to detect risk of suicide in an accurate, efficient, and timely manner. Although there is initial evidence for the efficacy of such an approach [4], current evidence lacks application to real-world ecologies and real-time assessment, both of which are essential if these insights are to move beyond the lab. Thus, we aimed to use artificial intelligence approaches to automatically classify in real time a large sample of telephone counselling calls made to Australian suicide-prevention help line services using voice biomarkers. By classifying counselling help line calls to a very high level of accuracy, we aim to demonstrate a viable support to existing help line infrastructure that can be employed in real time.

## Methods

Multimedia Appendix 1 illustrates the analysis workflow.

### Call Recordings

A total of 532 telephone call recordings were sourced for this retrospective observational study. Of these, 77 (14.5%) featured imminent risk of suicide, while 204 (38.3%) featured low risk of suicide. Participants were callers of Suicide Call-Back Service (a national help line service coordinated by On The Line, Australia) and 000 Emergency Services, Canberra wishing to discuss themes relevant to suicide risk. On The Line call recordings (n=517, 97.2%) were randomly sampled from July 1, 2019, to June 30, 2021, stratified by organizationally determined suicide risk level and disclosed sex of caller. In the case of 000 calls (n=15, 2.8%), call recordings were randomly sampled over the same time to reflect callers exhibiting imminent risk of suicide, necessitating emergency services’ response. Moderate-risk calls (236/517, 45.6%; Columbia Suicide Severity Risk ratings= 3 or 4) were removed from further analysis as they were not relevant to the aims of the study.

### Ethics Approval

No contact information for callers was possible, and a waiver of consent was granted by the Swinburne University Human Research Ethics Committee (reference number: 2021-4340). This study is reported in accordance with the CONSORT (Consolidated Standards of Reporting Trials) checklist [16]. A CONSORT attrition flowchart is provided in Multimedia Appendix 2. This study was registered with the Australian and New Zealand Clinical Trials Registry (ACTRN12622000486729) [17].

### Preprocessing of Calls

All calls were recorded in monochannel 8-kHz, 32-bit float format. Preprocessing involved transformation to 16-bit pulse-code modulation format, normalization, and pre-emphasis, which attenuated low signals and emphasized higher frequency signals to clarify the degree of audibility. This was important to reduce the effect of background noise. Listwise removal of silent frames (1,283,286/1,752,618, 73.2% speech frames) was performed prior to the following analyses.

### Selection of Low Versus Imminent Risk of Suicide Calls for Analysis

A multigated approach informed the designation of suicide risk level. Imminent risk of suicide was confirmed via affirmative responses (by the caller) to the following 3 screening questions: “are you having thoughts of suicide?”, “do you have a plan?”, and “are the means available?”, in compliance with triage guidelines [11]. The level of risk was then reassessed at the conclusion of each call by the responding counsellor using an organizationally developed framework; 6-point Likert-style scale (0-1=low; 2-3=medium; and 4-6=high). Responding counsellors also made clinical notes (eg, presentation of important content), which were inspected to ensure good correspondence to the assigned levels of suicide risk.

The level of risk for each call was then reassessed by a team of associate researchers (n=6), blinded to the initial rating. The associate researchers were psychologists either provisionally or fully registered with the Psychology Board of Australia, who had substantial prior experience working with suicidal presentations in telehealth settings. A random sample of calls (n=100) was provided to each researcher for reassessment using the Columbia Suicide Severity Rating Scale, a validated measure of suicide risk when used by clinicians [18] and administered via telephone [19]. Interrater reliability (kappa) of the Suicide Severity Rating Scale among the team of 6 associate researchers was 0.92 for a random selection of 12 recordings. The researchers were also asked to annotate segments of each recording using appropriate audio software (Audacity, Version 2.4.2; Audacity Team). Annotated segments of each recording were to be free from the counsellors’ voice as much as possible. Each annotated segment of speech was also described using mental status examination language.

### Derivation of Voice Biomarkers for Identifying Imminent-Risk Calls

Each annotated sound segment was divided into 50% overlapping 40 milliseconds Blackman-filter windowed frames [20]. The frame size ensured an adequate level of magnification of important characteristics at the center of the frame, while the degree of overlap ensured that the tails of each window did not remove valuable information. The modelling of risk occurred via 36 different voice biomarkers. Voice biomarkers are defined at both the 40 milliseconds speech frame and segment levels in the generalized additive mixed effects regression model described below.

### Reduction of Voice Biomarkers Using Penalized Lasso Regression

Penalized Lasso regression [21] was performed in the first instance to reduce the number of possible predictors to only those with a strong relationship with suicide risk. However, this model assumes linear relationships between predictors and response (conveyed via a logit link function) and ignores gender effects and correlations among segments across a single call. Thus, this model was used primarily to reduce the set of predictors that informed subsequent analyses.

### Validation via Mixed Effects Generalized Linear Regression

A 3-level model best reflected the approach to data collection. This model was used to confirm the significance of the reduced predictor set and test for significant moderation by caller sex, while allowing for the correlation between speech frames within each call. Model variables are summarized in Multimedia Appendix 3.

Splines were applied to each biomarker to account for nonlinearity [22]. Random intercepts at level 3 accounted for differences between individual calls, and a binomial model with logit link was used to identify imminent risk speech frames in terms of the level 1 and 2 voice biomarkers.

Without a comparable prior study, a power analysis for the final classification algorithm was not feasible. However, Pestian and colleagues [5] were able to achieve levels of classification accuracy of 85% with 371 recordings (level 2) and ~15 voice biomarkers (level 1). With a more precise classification model (component-wise gradient boosting) and a mixture of level 1 and level 2 predictors, we anticipated that a smaller sample size would suffice for this new study.

### Classification of Calls Using a Gradient Boosting Classification Model

Although powerful, support vector machines, as used by Pestian and colleagues [5], have notable disadvantages. Computation time is prohibitive when the data set is large (eg, >100,000 observations) and the choice of kernel, which allows the algorithm to choose a path of demarcation between groups while minimizing misclassification error, can be difficult. This is especially true when there is little to guide the choice of kernel, which is the case in analysis problems concerning voice biometrics. Finally, the mathematical complexity of support vector machines reduces the transparency of classification decision-making.

In comparison to support vector machines, gradient boosting is a computationally simpler approach that addresses many of the aforementioned problems. However, in its base implementation, it assumes linearity among the predictors. This can be remedied with an alternative implementation. Component-wise gradient boosting can analyze nonlinear data by first estimating a generalized additive mixed model with splines added, and then applying each model component (individual predictors and random components) to achieve the best reduction in classification error (eg, see Hofner [23] for a detailed overview). It is an approach that also allows for sex-moderated effects for all biomarkers.

Leave-one-out cross validation was used to test the classification accuracy of the gamboost model and to prevent information leakage occurring if data from one participant was used in both the training and test data sets. Thus, n–1 callers were used to train the model, leaving the nested speech frames of a single caller as the test case, ensuring independence of data between training and test data sets. Classification probabilities were derived for each speech frame (40 milliseconds) within each hold-out caller. Frame level classification probabilities were summarized by the mean classification probability for each hold-out caller.

The Youden J index was used to derive the ideal cut point that maximized upon both sensitivity and specificity of the classification accuracy across all hold-out callers in relation to binary precoded suicide risk level. A total of 1000 bootstrap samples were estimated, and the mean of these estimated samples was used as the ideal cut point. This approach minimizes sample-specific bias and possible overestimation of diagnostic utility, as discussed in Thiele and Herschfeld [24], and is an approach used by other authors, such as Hentschel [25].

Overfitting is suggested when there is a drop in classification accuracy between training and validation classification accuracy, suggesting that the algorithm has *memorized* the basis for classification and applies these insights poorly to new data. Classification accuracy was determined via accuracy measures including area under the receiver operating characteristic and area under the precision-recall curve. Plain language descriptions of all voice biometrics are contained in Multimedia Appendix 4.

## Results

Select caller demographics are summarized in Multimedia Appendix 5. The sample comprised 77/281 (27.4%) callers at imminent risk of suicide and 204/281 (72.6%) at low risk of suicide (n=87, 40% male callers and n=194, 70% female callers). Voice biomarkers were derived and analyzed for each of the 470,032 forty-millisecond speech frames. Median number of annotated segments per recording was 13.0 (SD 14.58), and median length of each segment was 118.50 (SD 120.19) milliseconds.

### Reduction of Voice Biomarkers Using Penalized Lasso Regression

Penalized Lasso regression was performed to reduce the number of voice biomarkers used to predict imminent suicide. A total of 36 initial voice biomarkers were reduced to 12. The significant predictors are summarized in Multimedia Appendix 6.

### Validation via Mixed Effects Generalized Linear Regression

A generalized additive mixed model was employed to validate the 12 predictors chosen by the Lasso regression. The model explained 12.0% (adjusted R^2^) of the variance in risk level at the segment level (N=3070 annotated segments).

**Figure 1:**
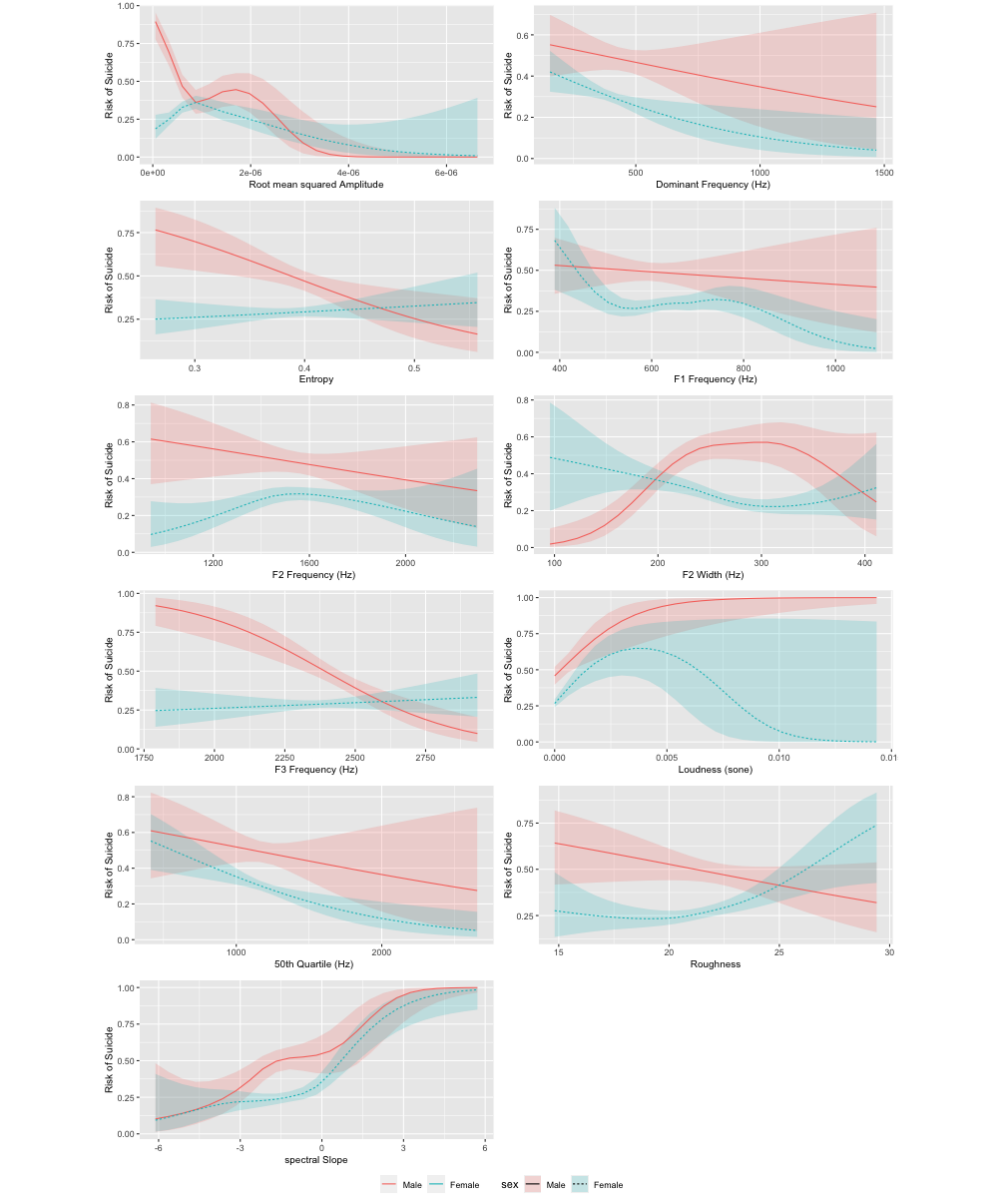
Plots of generalized additive mixed effects model predictors; nonlinear relationship between voice biomarkers and the risk of suicide with 95% CIs.

Table 1 summarizes the significance of spline coefficients in the generalized additive mixed model. Sex of caller was a significant moderator. The effective degrees of freedom indicate the degree of nonlinearity for each voice biomarker, with higher effective degrees of freedom indicating a greater degree of nonlinearity, and effective degrees of freedom close to 1 indicating linearity. Figure 1 illustrates the relationship between each voice biomarkers and the probability of imminent suicide, separately for male and female callers. For example, the plot of root mean squared amplitude suggests that both male and female callers speak with less signal strength (conceptually analogous to speaking in hushed tones) when at imminent risk of suicide. Conversely, increases in spectral slope were observed in both male and female callers as the level of risk of suicide increased, suggesting an increase in physiological effort when experiencing increasing suicidal stress.

These results confirmed that 11 (92%) of the 12 voice biomarkers were significant predictors of imminent suicide risk. First formant frequency, which proved nonsignificant for both male and female callers, was not included in the subsequent Component-wise gradient boosting model.

**Table 1:** Voice biomarker significance in the prediction of suicide risk: generalized additive mixed model^a^ (adjusted R2=0.12; N=3070).

Voice biomarker significance		*β*	SE	95% CI	EDF^b^	*F*-test	*P* value
**Male**							
	Root mean squared amplitude (dB)	–19.02	6.91	(–19.26, –18.77)	4.23	10.99	<.001
	Dominant frequency (Hz)	–1.10	1.10	(–1.14, –1.07)	1.00	1.00	.32
	Entropy	–2.21	0.80	(–2.24, –2.18)	1.00	7.56	.006
	Formant_1_ frequency (Hz)	–0.42	0.89	(–0.45, –0.39)	1.00	0.23	.63
	Formant_1_ width (Hz)	–0.60	1.28	(–0.65, –0.56)	2.46	3.05	.06
	Formant_2_ frequency (Hz)	–0.88	0.84	(–0.91, –0.85)	1.00	1.11	.29
	Formant_2_ width (Hz)	2.09	0.86	(2.06, 2.12)	3.57	10.16	<.001
	Formant_3_ frequency (Hz)	–3.61	0.76	(–3.64, –3.59)	1.00	22.68	<.001
	Loudness	8.57	2.79	(8.47, 8.67)	1.00	9.52	<.001
	50th quartile (Hz)	–1.14	1.27	(–1.19, –1.10)	1.03	0.82	.37
	Roughness	–1.05	0.71	(–1.07, –1.02)	1.00	2.21	.12
	Spectral slope	7.60	2.09	(7.53, 7.67)	4.10	4.76	<.001
**Female**							
	Root mean squared amplitude (dB)	–3.67	2.28	(–3.75, –3.59)	3.74	7.11	<.001
	Dominant frequency (Hz)	–2.41	0.90	(–2.44, –2.37)	1.02	6.63	.01
	Entropy	0.36	0.49	(0.34, 0.38)	1.00	0.54	.47
	Formant_1_ frequency (Hz)	–3.09	1.10	(–3.13, –3.05)	4.34	2.71	.02
	Formant_1_ width (Hz)	3.28	1.35	(3.23, 3.32)	3.44	2.00	.07
	Formant_2_ frequency (Hz)	0.17	0.76	(0.14, 0.19)	2.79	4.18	.02
	Formant_2_ width (Hz)	–0.74	0.58	(–0.76, –0.72)	2.90	5.52	<.001
	Formant_3_ frequency (Hz)	0.32	0.50	(0.30, 0.34)	1.01	0.39	.53
	Loudness	–5.97	4.55	(–6.13, –5.81)	1.91	8.34	<.001
	50th quartile (Hz)	–2.53	0.74	(–2.56, –2.50)	1.03	10.56	<.001
	Roughness	1.62	0.62	(1.60, 1.64)	2.58	4.65	.005
	Spectral slope	4.99	1.20	(4.94, 5.03)	4.10	10.53	<.001

^a^Male versus female: *β*=–.84; SE 0.002, 95% CI (–0.85, –0.84), *t*=6.59 (2 tailed); *P*<.001.

^b^EDF: effective degrees of freedom.

### Classification of Calls Using a Gradient Boosting Classification Model

Component-wise gradient boosting was used to classify each speech frame in terms of low and imminent risk of suicide. Leave-one-out cross-validation was used to test the classification accuracy of the gamboost model. A Youden J index value of 0.51 optimized upon both sensitivity and specificity in the classification of imminent risk. We correctly classified 469,332/470,032 (99.85%) speech frames (area under the receiver operating characteristic=1.0, 95% CI 1.0-1.0; area under the precision-recall curve=0.989, 95% CI 0.97-1.00).

While all low–suicide-risk speech frames were correctly classified, 700 (0.53%) of the 132,741 imminent risk frames were misclassified as low. This corresponded with the speech frames of a single caller in the 000 Emergency Services, Canberra sample. Upon closer inspection, this caller presented in an intoxicated manner, having ingested a “large amount of sleeping tablets.” Mental status examination annotations made by the reviewing team of psychologists indicated this caller spoke with slow-to-normal rate of speech and flat-to-neutral affect, and was responsive to all questions asked, a presentation similar to many low–suicide-risk callers.

## Discussion

The development of a timely and accurate form of suicide risk assessment remains a significant challenge, especially if implemented in a real-time capacity as required by suicide-prevention help lines. In this study, we sought to automatically classify short segments of speech obtained from 2 suicide-prevention telehealth services in Australia, according to low and imminent risk of suicide using supervised machine learning approaches. We successfully classified 469,332/470,032 (99.85%) speech frames, with only a small number (700/132,741, 0.53%) of high-risk speech frames misclassified.

Our study compares favorably with the findings of Pestian and colleagues [5], who successfully classified 322 (85.0%) of 379 participant recordings discriminating between low and high risk of suicide, using support vector machines. However, our study differs in a number of important ways from the aforementioned study. Rather than classify suicide risk at the holistic recording level, we instead classified risk at the 40 milliseconds frame level. This allowed us to expand upon the size of the data set upon which the classification algorithm could be trained and validated, allowing for a more nuanced assessment of each voice biomarker. This approach also demonstrates that only a short segment of a call is required for suicide risk classification, suggesting that the algorithm can be used for triage purposes based on only a short exchange (eg, an exchange with a triaging chatbot).

Second, we achieved a greater level of transparency and refinement than was afforded by the support vector machine in the study by Pestian and colleagues [5]. Figure 1 illustrates the exact nature of the relationship between the 11 voice biomarkers and the level of risk of suicide for men and women and suggests several discernible nuances in the ways male and female callers might speak when experiencing suicidal stress.

Finally, we reduced the numbers of possible predictors (via Lasso regression) to ensure that only the most statistically relevant biomarkers were included in later models. Our choice of generalized additive mixed model validated the use of all but one of the voice biomarkers selected by the Lasso regression.

However, there are limitations in our approach. We did not include callers of minority status, such as members of the LGBTIQ+ (lesbian, gay, bisexual, transgender, intersex, queer, and other people of diverse sexuality, gender, or bodily characteristics) communities and callers of non–English speaking backgrounds. These community members may offer valuable information that can further enhance the diversity of input, practical outcomes, and nuance of our analyses overall.

We were also not always successful in classifying all calls in the imminent suicide risk category. The similarity between the presentations of the single misclassified imminent risk caller and other low suicide risk callers suggests a possible subsample of callers who may be in the midst of a suicide attempt that is not being recognized by our classification approach. This is of concern given this presentation is most at need of timely emergency support. This suggests a possible role for other forms of classification such as natural language processing of speech-to-text translation, which might reduce similar misclassifications in the future.

There were also notable strengths in our approach. Our industry partnerships with On The Line, Australia and the Australian Federal Police, Canberra ensured that we could trial this novel technology in ecologically valid settings, where call quality is often degraded and background noise evident. This contrasts with the clinical settings within which the majority of studies have thus far been conducted. Our multigated approach to the assignment of suicide risk to each call ensured the establishment of a credible ground truth that was pivotal in accurately training the classification algorithm. Our choice of advanced statistical modelling has ensured a robust account of error variance in estimating the probability of imminent suicide. A final strength of this study is the visualization of imminent risk of suicide in terms of voice biomarkers allowing for nonlinearity.

This study has taken evidence from 25 years of pilot research and extended it to real world scenarios involving present-moment intent to suicide. However, it would be beneficial to control for caller age. We did not control for age in any of our analyses; however, given the well-documented age-related changes in vocal quality, this should feature in subsequent analyses and would boost an account of variance achieved by future mixed effects modelling. Finally, these compelling findings suggest possible implementation within a suicide-prevention telehealth service as an avenue worthy of further exploration.

## Appendix

Multimedia Appendix 1 Analysis workflow.

Multimedia Appendix 2 CONSORT (Consolidated Standards of Reporting Trials) attrition flowchart.

Multimedia Appendix 3 Summary generalized additive mixed effect model predictors.

Multimedia Appendix 4 Definitions of voice biomarkers terminology.

Multimedia Appendix 5 Summary caller characteristics.

Multimedia Appendix 6 Summary of significant level 1 and level 2 Lasso regression predictors.

## Abbreviations

CONSORT: Consolidated Standards of Reporting Trials
LGBTIQ+: lesbian, gay, bisexual, transgender, intersex, queer, and other people of diverse sexuality, gender, or bodily characteristics
