# Supplementary figures and images for: Using Voice Biomarkers to Classify Suicide Risk in Adult Telehealth Callers: Retrospective Observational Study
Source: JMIR Ment Health. 2022 Aug 15;9(8):e39807. doi: 10.2196/39807 (PMC9425169; doi:10.2196/39807)

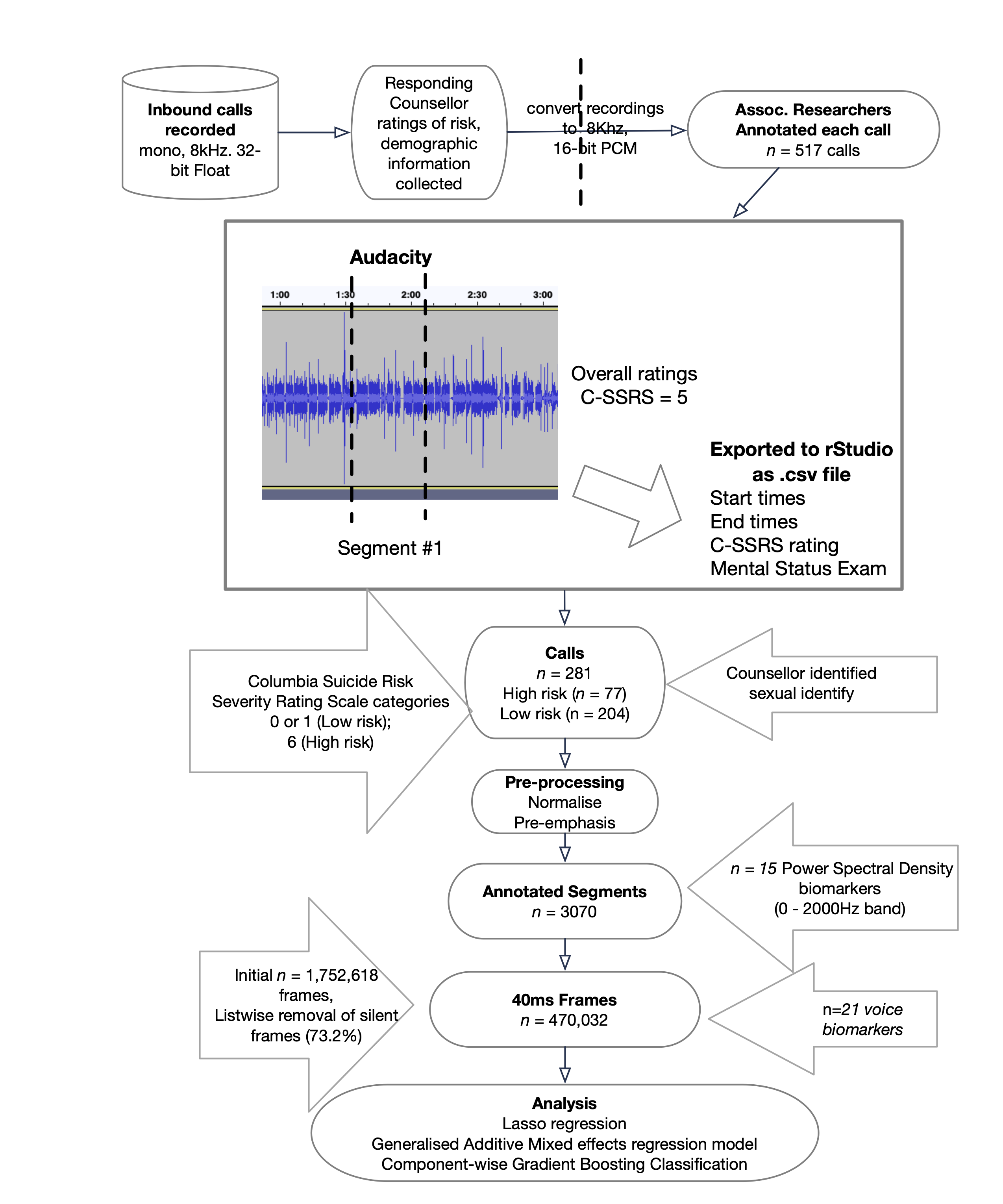

Supplement: Multimedia Appendix 1 [file mental_v9i8e39807_app1.png]

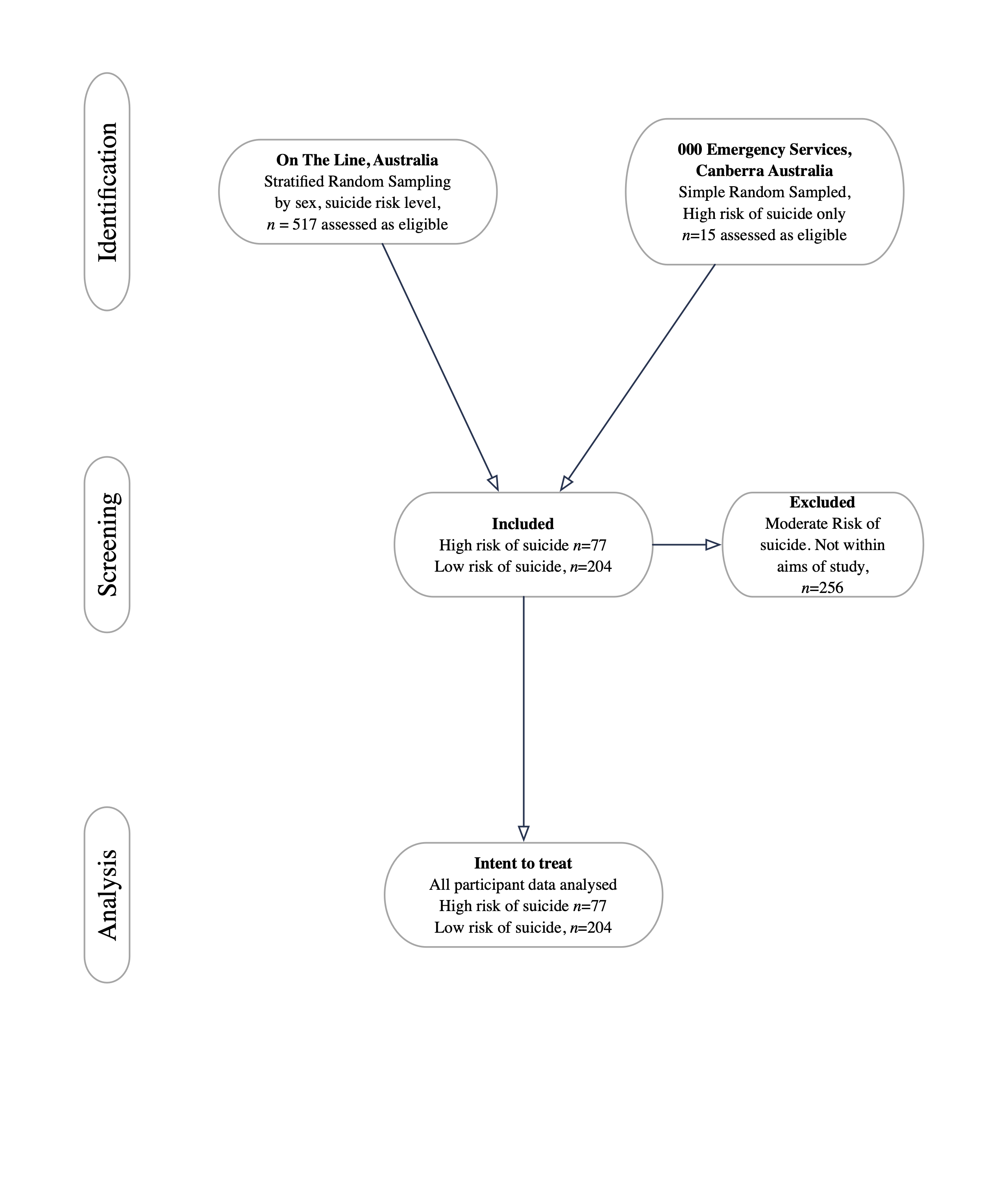

Supplement: Multimedia Appendix 2 [file mental_v9i8e39807_app2.png]
