# Supplementary material for: Using Voice Biomarkers to Classify Suicide Risk in Adult Telehealth Callers: Retrospective Observational Study
Source: JMIR Ment Health. 2022 Aug 15;9(8):e39807. doi: 10.2196/39807 (PMC9425169; doi:10.2196/39807)
Supplement: Multimedia Appendix 3 [file mental_v9i8e39807_app3.docx]

| Level | Variable Name |
| --- | --- |
| **Response Variable** |  |
|  | Low vs. High Suicide Risk |
| **Level 1 (21 variables per 40ms Speech Frame)** | |
|  | Root Mean Squared Amplitude (Hz) |
|  | Dominant Frequency (Hz) |
|  | Entropy (range = 0 – ∞) |
|  | Formant_1-3_ Frequency (Hz) |
|  | Formant_1-3_ Width (Hz) |
|  | Spectral Flux (range = 0 – ∞) |
|  | Harmonics/Noise Ratio (Range = 0 – ∞) |
|  | Loudness (Sone) |
|  | Spectral Novelty |
|  | Peak Frequency (Hz) |
|  | 25^th^/50^th^/75^th^ Quartile Frequency (Hz) |
|  | Roughness |
|  | Spectral Centroid (Hz) |
|  | Spectral Slope (dB/kHz) |
|  | Depth of Subharmonics per Frame |
| Autocorrelation (AR1) between frames was accounted for. | |
| **Level 2 (15 Variables per Annotated Segment; Power Spectral Density based, 0-2kHz)** | |
|  | Mean Frequency (Hz) |
|  | Standard Deviation of Mean Frequency (Hz) |
|  | Median frequency (Hz) |
|  | Standard error of the mean (Hz) |
|  | Most frequently occurring frequency (Hz) |
|  | 25^th^ Quartile of Frequencies (Hz) |
|  | 75^th^ Quartile of Frequencies (Hz) |
|  | Inter-Quartile Range (Hz) |
|  | Centroid (Hz) |
|  | Skewness (Hz) |
|  | kurtosis (Hz) |
|  | Spectral Flatness (range = 0 – 1) |
|  | Entropy |
|  | Measurement Precision |
|  | Number Sequential voiced/unvoiced/silence 40ms speech frames |
| **Level 3** **Individual callers** | |
|  | Sex Moderator Variable |
