# Supplementary material for: Using Voice Biomarkers to Classify Suicide Risk in Adult Telehealth Callers: Retrospective Observational Study
Source: JMIR Ment Health. 2022 Aug 15;9(8):e39807. doi: 10.2196/39807 (PMC9425169; doi:10.2196/39807)
Supplement: Multimedia Appendix 4 [file mental_v9i8e39807_app4.docx]

| Variable Name | Description |
| --- | --- |
| Root Mean Squared Amplitude (dB) | Root mean squared Amplitude. The mathematical operations ensure that both the negative and positive amplitude segments (either side of zero amplitude on a normal sound wave signal) are measured. Thus, it reflects the ‘absolute’ amplitude or linear loudness. |
| Dominant Frequency (Hz) | First, candidate pitch frequencies are estimated using the modulation spectrum (see below). The Lowest Dominant Frequency reflects the lowest candidate pitch frequency found in the modulation spectrum. |
| Entropy | Measured on a scale of 0 – 1, where 0 suggests that the energy in the signal is held within the upper harmonics, while 1 suggests the signal is more akin to white noise. Thus, Entropy is a measure of signal quality. |
| Formant_n_ Frequency (Hz) | The frequency locus of the n^th^ formant (see below). |
| Formant_n_ Width (Hz) | The frequency width of the locus of the n^th^ formant (see below). |
| Formant | It is a concentration of energy around a particular frequency. There are typically >4 formants identified in a spectrum, with the energy of each formant decreasing above 4. |
| Harmonics Energy | The ratio of energy held in the upper harmonics (>1.25*Dom [see above]) versus the energy held in the lower harmonics (<1.25*Dom).  Measured in dB. |
| Harmonics Height | How high do the upper harmonics reach in the modulation spectrum (see below). Measured in dB. |
| Harmonics/Noise Ratio | A measure of quantity of energy held in the harmonics compared to noise. Thus, 0dB suggests that equivalent amounts of energy are held jointly within the harmonics and in noise, while >0dB suggests increasingly greater amounts of energy are held within the harmonics. |
| Loudness | A measure of subjective loudness, measured in Sone, where 1 Sone = ~ 1000Hz. Measured on log scale to simulate human hearing |
| Modulation Spectrum | A translation (or decomposition) of a normal sound wave signal into a two-dimensional representation of frequencies (Hz) on the y-axis versus frequency (quantity) over time on the x-axis. |
| Novelty | A measure of variability in the modulation spectrum over time. |
| Peak Frequency (Hz) | The frequency with a maximum energy encountered in a modulation spectrum. Measured in Hz. |
| Power Spectral Density (PSD) | Refers to the distribution of power within the modulation spectrum (see above) over time. It is also common to divide the overall PSD into specific bandwidths e.g. 500Hz bands. |
| 25^th^/50^th^/75^th^ Quartile Frequency (Hz) | The frequencies corresponding with the quantiles of the modulation spectrum (see above). |
| Roughness | A measure of sound clarity. Higher values signify increasing roughness. Also an objective marker of dysphonia (see CPP above). |
| Spectral Centroid | A measure of how high the frequencies in a spectrum are on average. Refers to the listener’s average frequency that is heard. Measured in Hz. |
| Spectral Slope | The slope of the linear regression line of best fit of the frequencies in the modulation spectrum (see above). Increasing slope coefficients signify increasing physiological effort. |
| Depth of Subharmonics | Estimated depth of the subharmonics |
| Timing | The number of sequential voiced/unvoiced/silence 40ms frames in an annotated segment. |
