# Supplementary material for: Using Voice Biomarkers to Classify Suicide Risk in Adult Telehealth Callers: Retrospective Observational Study
Source: JMIR Ment Health. 2022 Aug 15;9(8):e39807. doi: 10.2196/39807 (PMC9425169; doi:10.2196/39807)
Supplement: Multimedia Appendix 5 [file mental_v9i8e39807_app5.docx]

|  |  | Suicide Call Back Service (*n*=517) | 000 Emergency (*n*=15) |
| --- | --- | --- | --- |
| **Suicide risk level (C-SSRS^a^)** | | |  |
|  | High | 62 | 15 |
|  | Medium^b^ | 236 |  |
|  | Low | 204 |  |
| **Sex** | |  |  |
|  | Female | 328 | 9 |
|  | Male | 159 | 6 |
|  | Transgender^b^ | 4 |  |
|  | Other^b^ | 2 |  |
|  | Unknown^b^ | 9 |  |
| **Age** | |  |  |
|  | 15-24 | 65 |  |
|  | 25-34 | 121 |  |
|  | 35-44 | 127 |  |
|  | 45-54 | 90 |  |
|  | 55-64 | 50 |  |
|  | 65+ | 34 |  |
| **Call time** | |  |  |
|  | 0:00-5:59 | 49 |  |
|  | 06:00-11:59 | 74 |  |
|  | 12:00-17:59 | 164 |  |
|  | 18:00-23:59 | 215 |  |

^a^ Columbia Suicide Severity Rating Scale

^b^ Excluded from the reported study
