# Supplementary material for: Using Voice Biomarkers to Classify Suicide Risk in Adult Telehealth Callers: Retrospective Observational Study
Source: JMIR Ment Health. 2022 Aug 15;9(8):e39807. doi: 10.2196/39807 (PMC9425169; doi:10.2196/39807)
Supplement: Multimedia Appendix 6 [file mental_v9i8e39807_app6.docx]

| Variables | | | Standardised *β* | | 95% C.I. | |
| --- | --- | --- | --- | --- | --- | --- |
| **Level 1 (40ms Speech Frames)** | | |  | |  | |
|  | Root Mean Squared Amplitude (dB) | -0.26 | | (-0.27, -0.25) | |  |
|  | Dominant Frequency (Hz) | 0.05 | | (0.05, -0.25) | |  |
|  | Entropy | 0.02 | | (0.03, -0.25) | |  |
|  | Formant_1_ Frequency (Hz) | -0.03 | | (-0.01, -0.25) | |  |
|  | Formant_1_ Width (Hz) | -0.06 | | (-0.05, -0.25) | |  |
|  | Formant_2_ Frequency (Hz) | -0.02 | | (-0.01, -0.25) | |  |
|  | Formant_2_ Width (Hz) | -0.04 | | (-0.04, -0.25) | |  |
|  | Formant_3_ Frequency (Hz) | -0.03 | | (-0.03, -0.25) | |  |
|  | Loudness (Sone) | 0.12 | | (0.12, 0.12) | |  |
|  | 50^th^ Quartile (Hz) | 0.04 | | (0.04, 0.04) | |  |
|  | Roughness | 0.05 | | (0.05, 0.05) | |  |
|  | Spectral Slope | 0.01 | | (0.06, -0.04) | |  |
|  |  |  | |  | |  |
| **Level 2 (Segments within Calls)** | | |  | |  | |
|  | Standard Error of Mean | 0.03 | | (0.03, 0.03) | |  |
|  | Spectral Entropy | -0.08 | | (-1.05, 0.89) | |  |
|  | Precision of Spectral Frequency | 0.04 | | (0.04, 0.04) | |  |
